# Exploiting and controlling gel-to-crystal transitions in multicomponent supramolecular gels†

**DOI:** 10.1039/d1sc02347k

**Published:** 2021-06-14

**Authors:** Demetra Giuri, Libby J. Marshall, Bart Dietrich, Daniel McDowall, Lisa Thomson, Jenny Y. Newton, Claire Wilson, Ralf Schweins, Dave J. Adams

**Affiliations:** Dipartimento di Chimica Giacomo Ciamician, Alma Mater Studiorum, Università di Bologna Via Selmi, 2 40126 Bologna Italy; School of Chemistry, University of Glasgow Glasgow G12 8QQ UK dave.adams@glasgow.ac.uk; Large Scale Structures Group, Institut Laue-Langevin 71 Avenue des Martyrs, CS 20156 F-38042 Grenoble CEDEX 9 France

## Abstract

Multicomponent supramolecular gels provide opportunities to form materials that are not accessible when using the single components alone. Different scenarios are possible when mixing multiple components, from complete co-assembly (mixing of the components within the self-assembled structures formed) to complete self-sorting such that each structure contains only one of the components. Most examples of multicomponent gels that currently exist form stable gels. Here, we show that this can be used to control the mechanical properties of the gels, but what is probably most exciting is that we show that we can use a magnetic field to control the shape of the crystals. The gelling component aligns in a magnetic field and so results in anisotropic crystals being formed.

## Abstract 2

Multicomponent supramolecular gels provide opportunities to form materials that are not accessible when using the single components alone.

## Graphical abstract

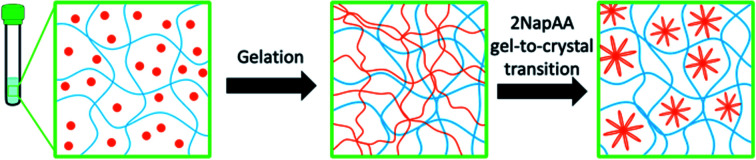

## Introduction

Supramolecular multicomponent gels are formed when multiple different molecules form a network that gives a system which is mainly liquid but has solid-like properties.^1–3^ A number of different types of system exist, but a powerful approach is to combine multiple small molecules that can each individually self-assemble to give a fibrous network. When combined, different outcomes are possible, from intimate mixing and co-assembly such that the fibrous structures contain all of the possible components to self-sorting, where fibrous structures are formed, but each is formed from just one of the components.^1^ Further complexity then arises from how these self-sorted fibres interact and entangle.^2^

Multicomponent systems can be used to form a range of interesting materials. Different components can be used to provide different properties, for example a structural and a cell-adhesion component,^4^ or alternatively to form the equivalent of a bulk heterojunction.^5–7^ A number of systems have been reported whereby specific properties have been enhanced in the mixture as compared to the single component equivalents. For example, the mechanical properties of the multicomponent systems can be stiffer than would be expected from the properties of the single component systems.^8,9^ The reason for such enhancement is not always clear.

Despite the potential of these systems, there are relatively few examples in the literature. Such systems are also much more complicated to characterize and understand. There are also limited methods to predetermine the type of combination that will result and, for example, effectively form self-sorted systems, with most relying on heat-cool cycles where there is limited predictability.

Functionalized dipeptides are one class of effective hydrogelator.^10–16^ Typically, such dipeptides can form gels using a pH switch.^11,12^ Here, a solution of the dipeptide is prepared at high pH (typically >10) resulting in the formation of a micellar dispersion. Decreasing the pH can result in gels.^11^ It is important here to control the rate of pH decrease if one wishes to form reproducible materials. We have shown that the slow hydrolysis of a lactone can be used to form gels with reproducible mechanical properties,^17^ and this slow hydrolysis also allows us to prepare self-sorted systems.^6,18,19^

Many of the dipeptides that can be used to prepare gels do so by forming long anisotropic structures such as cylindrical or elliptical fibres, which entangle and laterally associate to form the network.^11,12^ A very small number of dipeptides and functionalized dipeptides form gels from which crystals form over time.^20–26^ This gel-to-crystal transition results in the gel becoming weaker and eventually falling apart leading to phase separation into crystals and a solution. This can happen in single component systems, but also in multicomponent systems, where two different dipeptides are assembled in the presence of one another.^19^ Similar results have also been shown for other gelling systems.^27,28^

This gel-to-crystal transition has the potential for exploitation. Conceptually, it might be possible to induce different polymorph formation depending on the conditions used.^29–32^ Additionally, in multicomponent systems, this method can be used to change the properties of the gel phase and access materials with improved, unusual and interesting properties.^19,28^ Growing crystals in a dipeptide-based gel phase has been shown to protect the crystals against degradation.^33,34^ There is also the potential for a controlled ageing and weakening of the gel phase, and for a delayed release of particles embedded in the gel.

Here, we show how crystallization in a multicomponent system can be used to control the mechanical properties of the gels. There are differences in moving from H_2_O to D_2_O, which opens up opportunities to tune the system. We also show how an external magnetic field can be used to align the gelling component and how this directly affects the shape of the crystals formed from the second component.

## Results and discussion

**Fig. 1:**
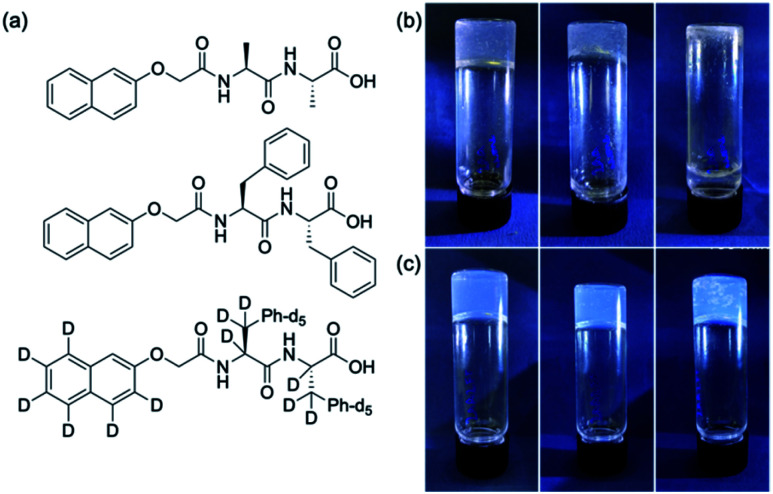
(a) The chemical structures of the two gelators used here (top) 2NapAA; (middle) 2NapFF; (bottom) the perdeuterated analogue of 2NapFF, 2dNapdFdF. (b) Photographs of the gels formed from 2NapAA at 15 min, 45 min and 180 min, with crystallisation from the gel phase leading to the gel falling apart. (c) Photographs of the gel formed from a mixture of 2NapAA and 2NapFF, at 15 min, 45 min and 180 min, with crystallisation occurring within the gel, but no overall loss of integrity of the gel.

We focus on the gels formed from two gelators, 2NapAA and 2NapFF (Fig. 1), as single and multicomponent gels. We have reported on both of these previously^21,35^ but our focus in this paper is exploiting the unusual behaviour exhibited by 2NapAA. 2NapAA is a rare example of such dipeptides which forms gels from which crystallization occurs over time.^21^ We show how this crystallization can be controlled and used to prepare multicomponent gels with increased rigidity. There are analogies here with polymer nanocomposites, where polymer films are reinforced with fillers.

At high pH, 2NapAA forms free-flowing solutions at a concentration of 5 mg mL^−1^. Decreasing the pH results in gelation, followed by crystallization, or direct crystallization depending on the rate of pH change and the final pH achieved. To decrease the pH, we use the hydrolysis of glucono-δ-lactone (GdL), which gives a slow and uniform pH change.^17^ The rate of pH change and final pH can be controlled by the amount of GdL added and there are also differences in the rate in H_2_O and D_2_O.^36^ Here, we use 20 mg mL^−1^ of GdL. Crystallization occurs in both H_2_O and D_2_O at this amount of GdL.

2NapFF alone forms viscous solutions at high pH and stable homogeneous gels at low pH as we have described elsewhere.^35^ We used a concentration of 2NapFF of 2.5 mg mL^−1^ as this gives relatively non-viscous solutions at high pH, meaning that mixing was straightforward. At this concentration, 2NapFF forms a transparent, homogeneous gel when GdL is added in both H_2_O and D_2_O which is stable with time.^37^

Mixing a 1 : 1 volumetric ratio of pre-formed solutions of 2NapAA (final concentration of 5 mg mL^−1^) and 2NapFF (final concentration of 2.5 mg mL^−1^) was carried out at high pH. 20 mg mL^−1^ GdL was then added to these solutions. These concentrations were chosen on the basis of optimising for rate of pH change, initial viscosity and the final rheology.

In the mixed system, a gel is initially formed. The formation of crystals starts around 20 minutes after the addition of the GdL in H_2_O and after 40 minutes in D_2_O. This can be seen from the optical microscope images (Fig. S5, ESI†). The crystals formed in these mixed systems have different shapes from the crystals formed by 2NapAA alone (Fig. S6 and S7†), with dendritic aggregates formed in the presence of 2NapFF. In D_2_O, the crystals start growing at a later time as compared to in H_2_O but become larger overall. Despite these visual differences, pXRD shows that the crystal structure is the same in all cases in these mixed gels as in the single component 2NapAA systems (Fig. S8, ESI†). Unlike the solution of 2NapAA alone, when crystals form, they do not sediment to the bottom of the vial; instead, the crystals are maintained within a stable gel network. We note that despite examining these mixtures under a number of different conditions, no sign of any other polymorph of 2NapAA has been observed.

**Fig. 2:**
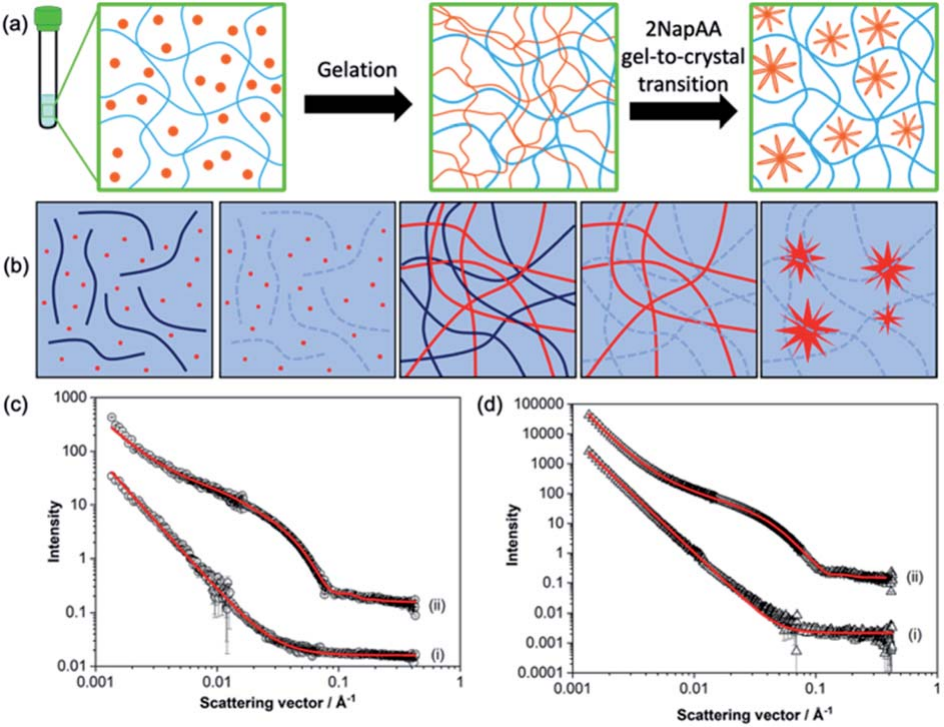
(a) Schematic showing the 2NapAA sol-to-gel-to-crystal transition in the presence of 2NapFF. 2NapAA is shown in orange and 2NapFF in blue. (b) Schematic showing how contrast matching can be used to differentiate between gelators in D_2_O. From left to right, at high pH where both gelators are hydrogenated; at high pH where the 2NapFF is deuterated; at low pH where both are hydrogenated; at low pH where the 2NapFF is deuterated; at low pH where the 2NapFF is deuterated and 2NapAA has crystallised. Where both gelators are hydrogenated, scattering comes from the structures formed from both gelators; where one of the gelators is deuterated, scattering only comes from the second component. (c) Small angle neutron scattering data from high pH solutions of the (i) 2NapAA : 2dNapdFdF mix and (ii) 2NapAA : 2NapFF in D_2_O. (d) Small angle neutron scattering data from gels formed from of (i) 2dNapdFdF : 2NapAA mix and (ii) 2NapFF : 2NapAA mix in D_2_O. In (c) and (d) black circles represent experimental data and red lines represent model fits from SasView. Intensity is dimensionless because the data are offset on the same *y* axis. The parameters for the fitting are shown in Table S1.†

A key question in such a mixture is whether the sample is initially forming mixed fibres from which the 2NapAA then crystallizes in some way, or self-sorted structures^1,2^ where each fibre contains only one of 2NapFF or 2NapAA. It is intuitively easier to see how 2NapAA would crystallize from such a system as the fibres would be analogous to those in a pure 2NapAA system (Fig. 2a).

However, here we have a complicated system where the micellar aggregates present at high pH may mix fully or partially and then mixing or self-sorting may persist or occur as the pH is decreased during gelation. We have previously shown for example that mixtures of 2NapFF with a second gelator can mix at high pH and partially mix on gelation depending on the ratios of gelators used.^38^

To answer this question, we prepared a perdeuterated analogue of 2NapFF, denoted 2dNapdFdF (Fig. 1c) and used small angle neutron scattering (SANS) to probe the self-assembled states at high pH (where micelles exist) and at low pH (the gel state). SANS was carried out in D_2_O to allow us to use contrast matching approaches to differentiate between the two gelators (Fig. 2b). We emphasize that these systems often exhibit drying artefacts and so imaging techniques such as TEM and SEM are not appropriate. Further, these only provide a small snapshot of the whole system. Scattering approaches allow us to understand the bulk sample. We used two systems, the mixture of 2NapAA and 2NapFF and the mixture of 2NapAA and 2dNapdFdF, a perdeuterated analogue of 2NapFF. In the first case, the scattering should come from both the 2NapFF and the 2NapAA. In the second case, the perdeuterated 2dNapdFdF should have little contrast as compared to the solvent (D_2_O) and hence the scattering should only come from the 2NapAA.^35,39^ The scattering for 2dNapdFdF alone at high pH in D_2_O is very low as expected (Fig. S9, ESI†). In H_2_O, where contrast from the deuterated 2dNapdFdF is expected,^39^ the scattering intensity is much higher and the data can best be fitted to a hollow cylinder combined with a power law, with a radius of 1.57 nm, a thickness of 2.24 nm and a length of 80 nm (Fig. 2c and Table S1, ESI†). These data are in close agreement with the data for 2NapFF in D_2_O,^35^ showing that deuteration of the molecule has not affected the self-assembly. In comparison, at high pH, 2NapAA alone in D_2_O scatters very weakly, which can be ascribed to the formation of transient micellar aggregates as we have described for related gelators.^35,40^

The mixed system of 2NapAA and 2dNapdFdF is weakly scattering (Fig. 2c), which is as expected if the two systems were acting independently as opposed to the 2NapAA being entrained within the 2dNapdFdF micelles (2NapAA scatters very weakly under these conditions). This demonstrates that there is self-sorting at high pH and the two components are operating independently.^2^ The scattering from 2NapAA and 2NapFF is best fitted to a cylinder and power law model (Fig. 2c and Table S1†). Crucially, there is a structural change from 2NapFF alone because there is no longer a hollow core (further discussion in ESI, Fig. S10 and Table S2†). These data suggest that although the 2NapAA influences the self-assembly of 2NapFF, it does not co-assemble with it.

These data are also backed up by further NMR data. 2NapFF micelles at high pH can be aligned in a magnetic field.^41^ The degree of alignment can be probed by the residual quadrupolar coupling to D_2_O.^38,41^ The data for 2NapFF alone and that for the mixture of 2NapAA and 2NapFF are within error margins (Fig. S12†), strongly suggesting that the micellar structures formed by 2NapFF are unaffected by the presence of 2NapAA. Hence, from the NMR and SANS data, this mixture is self-sorted in the micellar state at high pH (Fig. 2a).

Gels formed from just 2NapFF have previously been fitted to an elliptical cylinder and a power law.^35^ The SANS data for 2NapFF : 2NapAA mix gels in D_2_O were also best fitted to an elliptical cylinder with a power law (Fig. 2d and Table S1†). This suggests no significant change to self-assembled structures in the mixture in the gel phase as compared to 2NapFF alone and indicates self-sorting is occurring at low pH. The 2dNapdFdF : 2NapAA mix data show lower scattering intensity and was best fit to a power law consistent with the contrast matching of 2dNapdFdF and limited scattering from the 2NapAA. Limited scattering is seen from the 2NapAA as crystallization results in the formation of large structures that are outside the length scale accessible with SANS. If co-assembly occurred and the 2NapAA were entrained within the fibres of 2dNapdFdF, we would expect a scattering pattern consistent with a cylindrical structure. Instead, we see low scattering intensity that is best fitted to a power law, which is consistent with 2NapAA alone crystallising. The results therefore indicate self-sorted systems for both the 2NapAA : 2NapFF and 2NapAA : 2dNapdFdF mixtures in the gel phase.

For these dipeptide-based systems, self-sorting is most common in our experience when a slow pH trigger is applied. This can be explained by gelation occurring at the apparent p*K*_a_ of the aggregate of each dipeptide. We do observe co-assembly in some systems,^38^ but generally self-sorting occurs.^6,19^ We note that co-assembly has been suggested in other dipeptide systems^4^ and also is common for other gelling systems.^1,42^

**Fig. 3:**
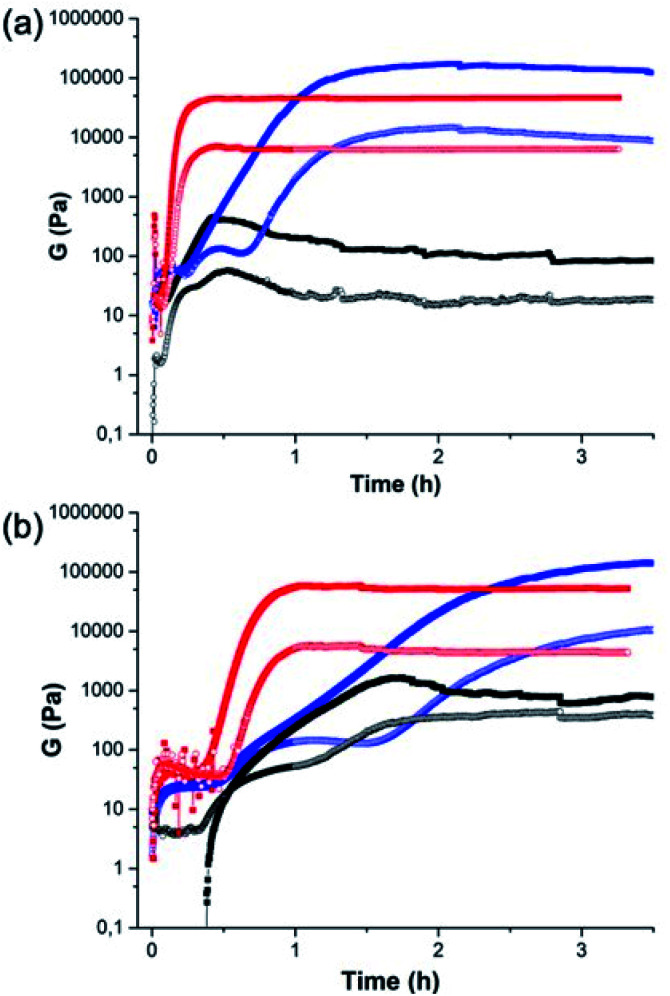
Comparison of the rheology between (black) 2NapAA; (red) 2NapFF; (blue) the mixture of 2NapAA and 2NapFF in (a) H_2_O and (b) D_2_O.

The crystallisation within the gel phase in a multicomponent system allows access to interesting material properties. From a rheological perspective, the mixed systems evolve differently as compared to the single component systems (Fig. 3). Fig. 3a shows the comparison of the time sweep rheology data for 2NapAA alone, 2NapFF alone, and the mixture of 2NapAA and 2NapFF in H_2_O. Solutions were also prepared and mixed in D_2_O (Fig. 3b). For 2NapAA, initially there is an increase in the storage and loss moduli (*G*′ and *G*′′ respectively), followed by a decrease in these values as crystallization occurs and noisy data at longer times. This process is slower in D_2_O as compared to H_2_O. In comparison, for 2NapFF alone, there is an increase in both *G*′ and *G*′′ with time, before a plateau is reached. At this point, as would be expected for a gel *G*′ dominates over *G*′′. Again, this process is slower in D_2_O as compared to H_2_O. For the mixed system, a gel is formed with an increase of both *G*′ and *G*′′ with time before a plateau is reached. In both H_2_O and D_2_O, gelation is slower in the mixed system as compared to the case of the 2NapFF alone. Whilst 2NapFF alone forms a gel where the moduli do not change significantly after around 1 hour in both H_2_O and D_2_O, in the mixed component system, a gel is formed more slowly, with the moduli decreasing slightly again at longer times. This is most pronounced in H_2_O and can be linked to the formation of the crystals from 2NapAA. The mixed component system is however significantly stiffer than either component alone. The differences in the behaviour in H_2_O and D_2_O can be ascribed to differences in the rate of GdL hydrolysis in H_2_O and D_2_O.^36^ Indeed, the overall rate of pH decrease is higher in H_2_O than in D_2_O for the mixture of 2NapAA and 2NapFF (Fig. S11†).

**Fig. 4:**
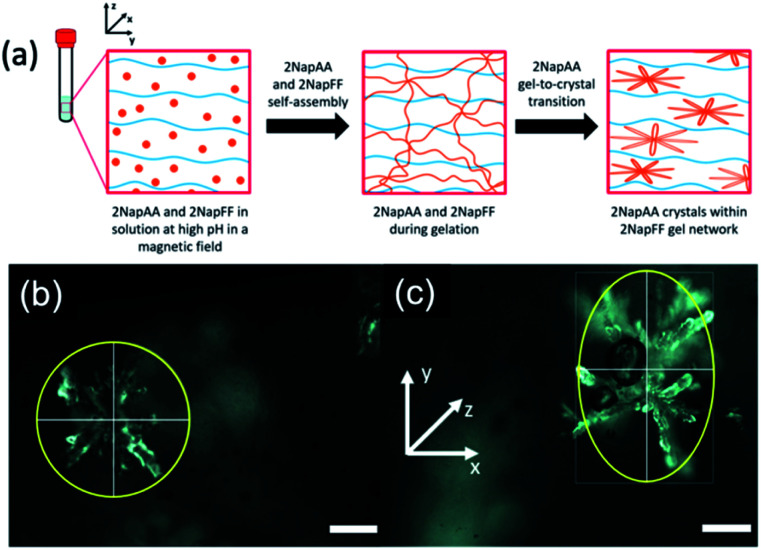
(a) Schematic showing the 2NapAA sol-to-gel-to-crystal transition in the presence of 2NapFF with a magnetic field. 2NapAA is shown in orange and 2NapFF in blue. Photographs of dendrites of crystals formed taken using an optical microscope (b) without and (c) with the presence of a magnetic field. The magnetic field lies along the *x*-axis, elongating the crystals in the *y*-direction of the magnetic field. Images were collected under polarized light and scale bars represent 300 μm.

Another interesting possibility in such systems is using the gelling component to influence the crystallization of the second component. For systems such as 2NapFF where worm-like micelles are formed at high pH, these can be aligned using a magnetic field. In comparison, the aggregates formed from dipeptides that are not anisotropic at high pH show no sign of alignment in a magnetic field at these pH values.^41^ Since 2NapFF can be aligned at high pH in a magnetic field (Fig. S12†), we hypothesized that it would therefore be possible to crystallize 2NapAA in an aligned media, which should lead to preferred orientation of the dendritic crystals as compared to crystallization out of the field (Fig. 4a).

Here, we optimized the system and so used a concentration of 5 mg mL^−1^ for each gelator. A higher concentration of 2NapFF was used to ensure alignment.^41^ GdL was added immediately prior to placing the sample in the magnetic field; here, we are exploiting the rapid alignment,^41^ and relatively slow hydrolysis of GdL and hence pH decrease.^17,36^ Comparison of the crystals formed in the multicomponent gel with and without the magnetic field (Fig. 4b and c, S14 and Table S3, ESI†) clearly shows that there is an elongation of the dendritic structures in the alignment direction. The lengths of the dendrites were measured in both *x*- and *y*-directions. We calculated the average *Y*/*X* ratio for the crystals grown with and without the magnetic field. The dendrites grown without the magnetic field had an average *Y*/*X* of 1.092 ± 0.104, very close to 1, and are essentially spherical. However, when dendrites were grown in the presence of the magnetic field, the average *Y*/*X* increased to 1.849 ± 0.418 in the direction of the magnetic field, showing an almost two-fold increase in length in the *y*-direction. No effect on the crystal shape was observed when 2NapAA was crystallised alone in the magnetic field (Fig. S15, ESI†).

## Conclusions

In conclusion, we have shown a self-sorted system is formed from the two gelators chosen here. Self-sorting occurs at both high pH and low pH, which can be proven using contrast matching small angle neutron scattering studies. This approach is very powerful and provides a more detailed understanding than can be accessed using many other techniques. We have shown that the use of a magnetic field results in the alignment of the self-assembled structures formed by one of the gelators growing faster in one axis as compared to in the absence of the field. Drying of agarose gels has been shown elsewhere to be able to lead to a change in the shape of crystals grown in the network;^43^ our work here opens up opportunities that do not require drying and for crystallization in multicomponent systems and could also be applicable to non-gelling components. We anticipate therefore that this will be of utility in many areas. There are analogies here with polymer nanocomposites, where polymer films are reinforced with fillers. However, unlike the situation where clays or other additives are added directly and must be colloidally stable across the entire gelation regime, here we form the crystalline additives *in situ*. There may of course be advantages to either approach, but our method does allow us to prepare structures *in situ*, with the shape and size being controlled by rate of gelation and the presence of an external field. We also suggest that there may be opportunities here to prepare samples with gradients of structure and composition.

## Data availability

The ESI is comprehensive and contains all of the necessary data.

## Author contributions

Conceptualisation (DG, DA); data curation (DG, LJM, BD, DM, LT, CW, RS); formal analysis (DG, BD, DM, JYN, CW, RS, DA); funding acquisition (DG, DA); investigation (all); methodology (all); project administration (DA); supervision (DA); writing (all).

## Conflicts of interest

There are no conflicts to declare.

## Supplementary Material

SC-012-D1SC02347K-s001
