# Supplementary material for: Exploiting and controlling gel-to-crystal transitions in multicomponent supramolecular gels
Source: Chem Sci. 2021 Jun 14;12(28):9720–5. doi: 10.1039/d1sc02347k (PMC8293982; doi:10.1039/d1sc02347k)
Supplement: SC-012-D1SC02347K-s001 [file SC-012-D1SC02347K-s001.pdf]

Electronic Supplementary Material (ESI) for Chemical Science.
This journal is © The Royal Society of Chemistry 2021

Exploiting and Controlling Gel-to-Crystal Transitions in Multicomponent Supramolecular Gels

Demetra Giuri,[a] Libby Marshall,[b] Bart Dietrich,[b] Daniel McDowall,[b] Lisa Thomson,[b] Jenny Y. Newton,[b] Claire Wilson,[b] Ralf Schweins,[c] and Dave J. Adams[b,*]

a Dipartimento di Chimica Giacomo Ciamician, Alma Mater Studiorum, Università di Bologna, Via Selmi, 2, 40126, Bologna, Italy

b School of Chemistry, University of Glasgow, Glasgow, G12 8QQ, U.K.

c Large Scale Structures Group, Institut Laue-Langevin, 71 Avenue des Martyrs, CS 20156, F-38042 Grenoble, CEDEX 9, France

Supporting Information

Experimental

Materials. The two gelators 2NapAA and 2NapFF were synthesized as previously described.[1, 2] The synthesis of perdeuterated analogue of 2NapFF, denoted 2dNapdFdF is described below. Deionized water was used throughout to prepare the gelator solutions.

Ethyl (2*S*)-2-[(2*S*)-2-{2-[(3,4,5,6,7,8-d_6)naphthalen-2-yloxy]acetamido}-3-[(2,3,4,5,6-d_5)phenyl](2,3,3-d_3)propanamido]-3-[(2,3,4,5,6-d_5)phenyl](d_3)propanoate (**GA-001**)

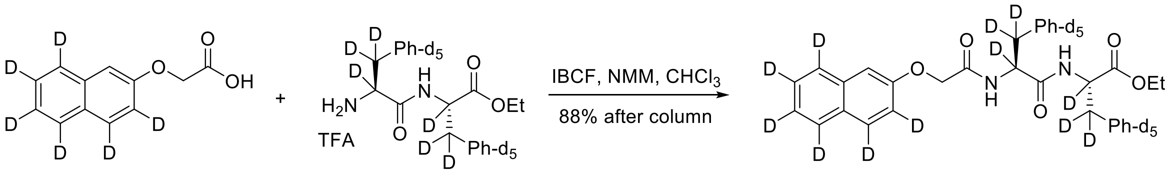

DA-001 and **EF-007** were prepared as previously described.[3] A solution of **DA-001** (319 mg, 1.53 mmol) and *N*-methylmorpholine (2.2 eq, 370 μL) in chloroform (20 mL) was cooled in ice/water. *Iso*butyl chloroformate (1.02 eq, 203 μL) was added followed by, 10 minutes later, **EF-007** (1.01 eq, 725 mg) and the mixture was allowed to reach room temperature overnight. After this time, it was diluted with chloroform, washed in turn with 1M hydrochloric acid, water, and brine, dried ($MgSO_4$) and evaporated to dryness under reduced pressure. Column chromatography (1:9 ethyl acetate/dichloromethane, *ca.* 4×3 cm, wet-loaded) afforded the title compound as an off-white solid (734 mg, 88%).

δ_H (400 MHz, DMSO-d_6) 8.60 (1H, s, NH), 8.15 (1H, s, NH), 7.19 (1H, s, naphthalene H-1), 4.54 (2H, s, OCH_2), 4.04 (2H, q, *J* 7.07, CH_2CH_3), 1.09 (3H, t, *J* 7.08, CH_2CH_3). δ_C (100 MHz, DMSO-d_6) 171.22, 170.98, and 167.25 (C=O), 155.44, 137.18, 136.64, 133.93, 129.22-128.39 (m), 128.58, 128.02-127.11 (m), 126.53-125.48 (m), 123.77-122.85 (m), 118.49-117.52 (m), and 107.29 (C_{Ar}), 66.69 (OCH_2), 60.49 (CH_2CH_3), 53.78-52.52 (m, CD*), 36.90-35.42 (m, CD_2), 13.89 (CH_2CH_3). HRMS (ESI) m/z: $[M+Na]^+$ calcd for $C_{32}H_{10}D_{22}N_2NaO_5$ 569.3584; found 569.3584. Mass spec data suggests presence of some $C_{32}H_{11}D_{21}N_2NaO_5$, i.e., one deuteron replaced by a proton. This would presumably be as a consequence of partial racemisation around one of the stereocenters.

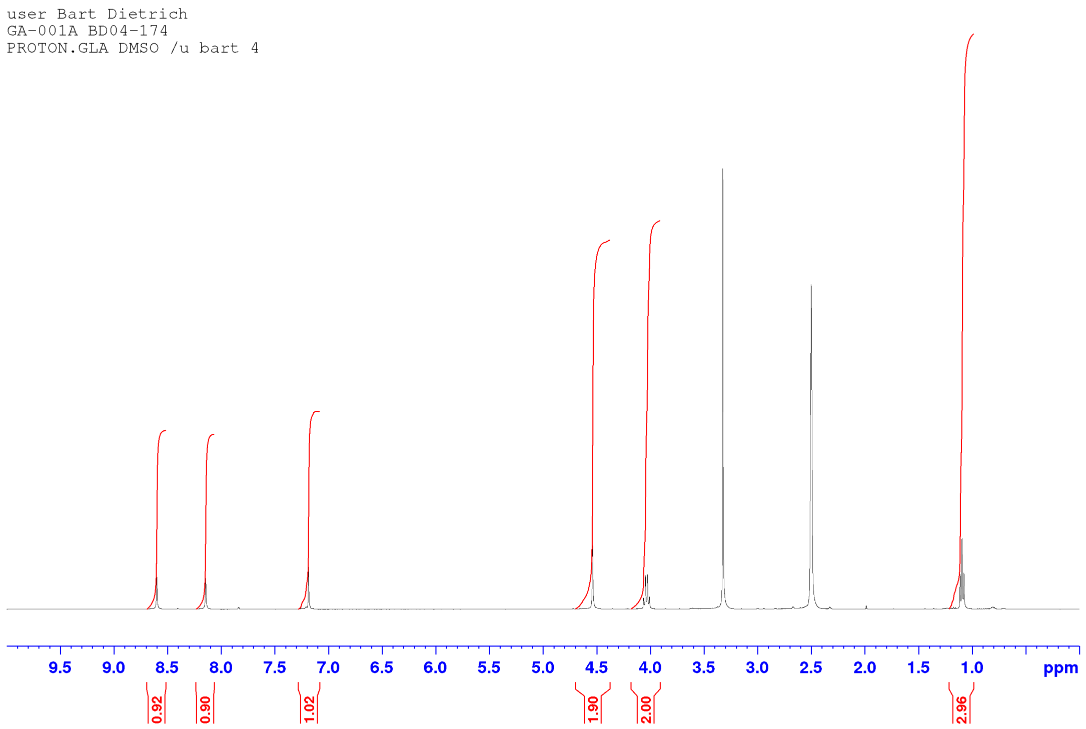

Figure S1. Proton NMR spectrum of **GA-001** in d_6-DMSO.

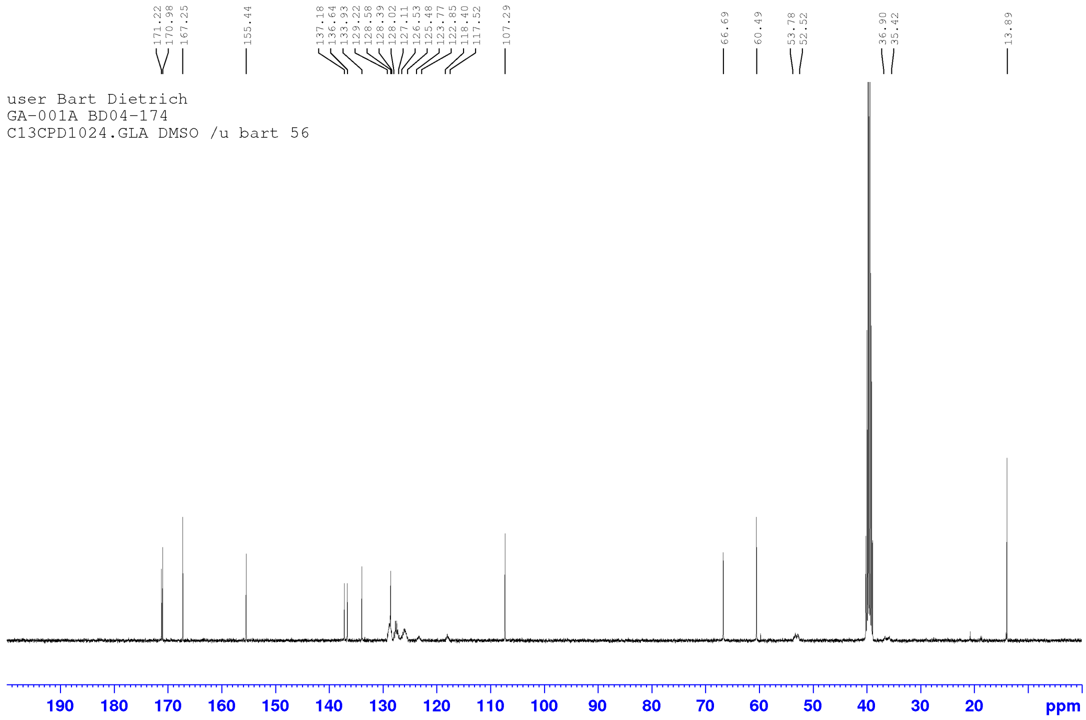

Figure S2. Carbon NMR spectrum of **GA-001** in d_6-DMSO.

(2*S*)-2-[(2*S*)-2-{2-[(3,4,5,6,7,8-d_6)Naphthalen-2-yloxy]acetamido}-3-[(2,3,4,5,6-d_5)phenyl](2,3,3-d_3)propanamido]-3-[(2,3,4,5,6-d_5)phenyl](d_3)propanoic acid (**GA-002**)

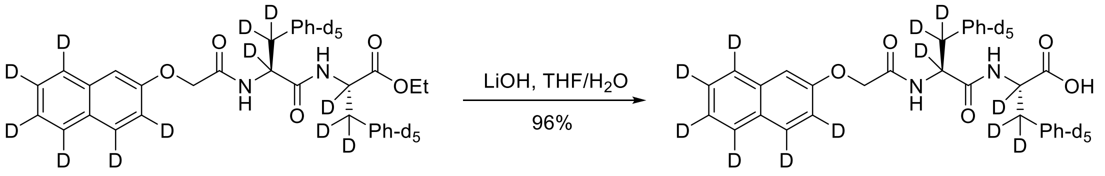

GA-001 **GA-002**

To a solution of **GA-001** (686 mg, 1.25 mmol) in tetrahydrofuran (8 mL) was added a solution of lithium hydroxide (4 eq, 120 mg) in water (8 mL). The mixture was stirred at ambient temperature for 4 hours then 1M hydrochloric acid (*ca.* 100 mL) was added. The mixture was stirred for an hour then filtered under suction. The solid in the filter was washed with several portions of water and allowed to partially dry under suction. It was then further dried by azeotropic distillation with acetonitrile affording the title compound as a white solid (620 mg, 96%).

δ_H (400 MHz, DMSO-d_6) 12.81 (1H, s br, COOH), 8.42 (1H, s, NH), 8.11 (1H, s, NH), 7.18 (1H, s, naphthalene H-1), 4.53 (2H, s, OCH$_2$). δ_C (100 MHz, DMSO-d_6) 172.72, 170.84, and 167.22 (C=O), 155.45, 137.21, 137.06, 133.94, 129.25-128.30 (m), 128.59, 128.06-126.89 (m), 126.45-125.32 (m), 123.80-122.86 (m), 118.52-117.68 (m), and 107.33 (C_{Ar}), 66.71 (OCH$_2$), 53.61-52.59 (m, CD*), 37.14-35.16 (CD$_2$). HRMS (ESI) m/z: $[M+Na]^+$ calcd for $C_{30}H_6D_{22}N_2NaO_5$ 541.3271; found 541.3256. Like in its precursor, the mass spectrum suggests the presence of some amount of $C_{30}H_7D_{21}N_2NaO_5$. The amount of this compound is presumed to be minimal since no proton NMR signal for it is observed.

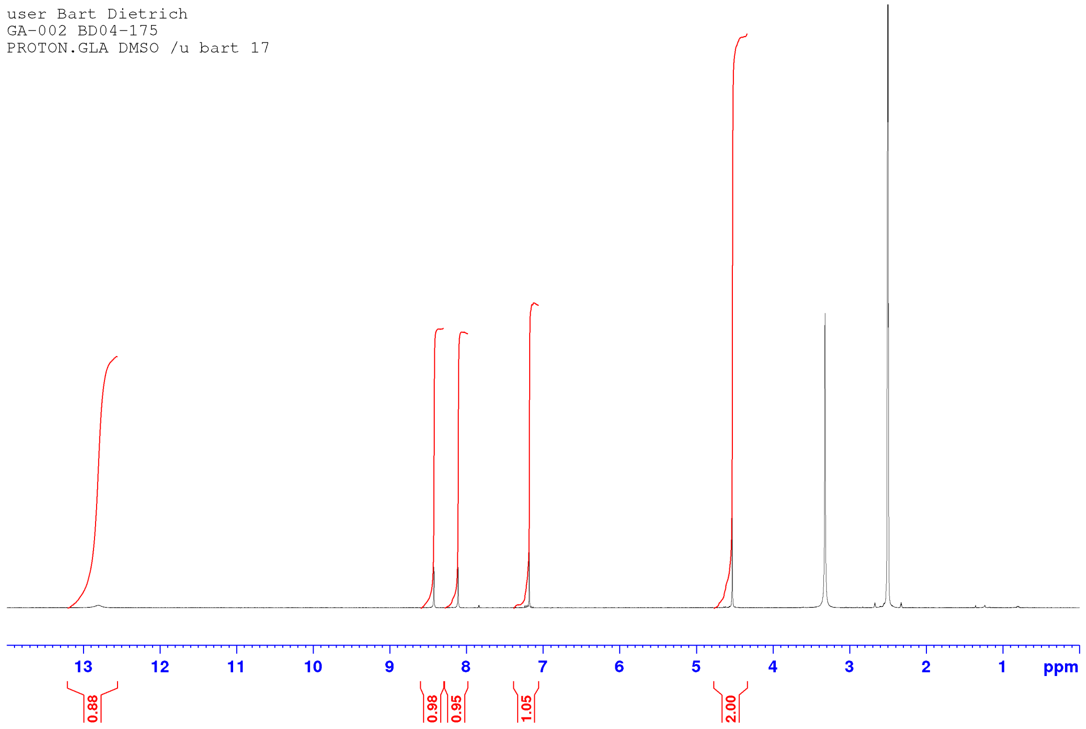

Figure S3. Proton NMR spectrum of **GA-002** in d_6-DMSO.

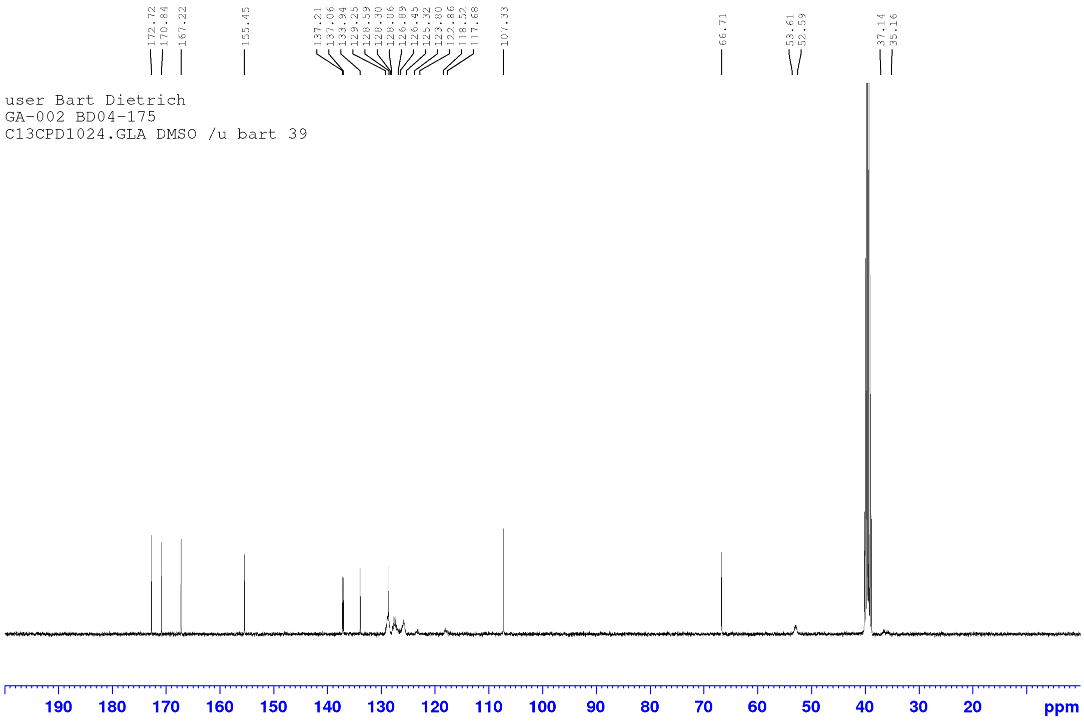

Figure S4. Carbon NMR spectrum of **GA-002** in d_6-DMSO.

Gelator Solutions. Stock solutions (20 mL) of 2NapAA (at a concentration of 5 mg/mL) and 2NapFF (at a concentration of 2.5 mg/mL) were prepared by suspending the desired gelator (100 mg for 2NapAA and 50 mg for 2NapFF) in deionized water (17.10 mL for 2NapAA and 19 mL for 2NapFF). An equimolar ratio of NaOH (0.1 M, aq.; 2.90 mL for 2NapAA and 1.00 mL for 2NapFF) was added, and the solutions were gently stirred until a clear solution was formed; the 2NapFF typically takes overnight to fully dissolve.

The pH of the solution was checked and adjusted to 10.5 if needed using NaOH (0.1 M). To form gels, 2 mL aliquots of either solution were placed in a 7 mL volume Sterilin vial containing a pre-weighed amount of glucono-δ-lactone (GdL). The samples were gently swirled to dissolve the GdL before being left to stand for 24 hours without stirring. After this time, the samples were examined by the vial inversion test to indicate gelation and any measurement was carried out at this point on a fresh sample for each measurement.

The solution of mixed gelators was prepared by dissolving both 2NapAA (such that the final concentration was 5 mg/mL) and 2NapFF (such that the final concentration was 2.5 mg/mL) in deionized water in the same vial and following the same procedure as for the single component systems.

Rheology. All rheological measurements were performed using an Anton Paar Physica MCR101 rheometer. A vane and cup measuring system was used, setting a gap of 1.8 mm. The gels were prepared as described above and tested directly in the Sterilin cup which fits in the rheometer. Time Sweep experiments were performed at 25 °C using a constant angular frequency (ω) of 10 rad/s and a constant shear stress (γ) of 0.5%.

pH control. The pH of the gels was monitored with time after GdL addition, placing the Sterilin vials in a pre-equilibrated water bath to maintain a constant temperature of 25 °C. A FC200 pH probe (HANNA instruments) with a (6 mm x 10 mm) conical tip was employed for the pH measurements with a stated accuracy of ± 0.1. pH changes during the gelation process were recorded every 15 seconds for 16 hours.

To log the pH, a custom-built pH and temperature measurement and logging device was used. This is compatible with standard BNC-terminated pH probes and is capable of recording pH/temperature data over hours or days and then exporting these data as an ASCII file for further processing.

Optical Microscopy. All samples were prepared in a CELLstar®TC (Greiner Bio-One, Stonehouse, UK) 35/10 mm plastic cell culture dish. 1 mL of the gelator solution was mixed with the desired quantity of GdL, mixed to ensure dissolution of the GdL and placed in the dish. This was covered to prevent drying issues.

Confocal Microscopy. All samples were prepared in a CELLview™ (Greiner Bio-One, Stonehouse, UK) 35/10 mm plastic cell culture dish with a borosilicate glass bottom. Confocal microscope images were taken using a Zeiss LSM710 confocal microscope. Nile Blue was added as dye (2 μL of 0.1% w/w solution for every mL of gelator solution). 1 mL of the gelator solution was mixed with the desired quantity of GdL, mixed to ensure dissolution of the GdL and 0.4 mL of the solution placed in the dish. Fluorescence from Nile blue was excited using a 633 nm helium neon laser and emission was detected above 650 nm.

Single crystal X-ray diffraction. Single crystal x-ray diffraction data were collected using Bruker D8 Venture equipped with Photon II CPAD detector, dual IμS 3.0 Cu and Mo sources.

Powder X-ray diffraction. PXRD (powder -ray diffraction) patterns were collected using a Rigaku MiniFlex 6G equipped with a D/teX Ultra detector, a 6-position (ASC-6) sample changer and Cu sealed tube ($K\alpha1$ and $K\alpha2$ wavelengths - 1.5406 and 1.5444 Å respectively). Patterns were measured as $\theta/2\theta$ scans typically over a range of $3>2\theta>60°$. Data collection and analysis were carried out using Rigaku SmartLab Studio II software (Rigaku Corporation, 2014).

The structure was determined from diffraction data collected at room temperature to facilitate comparison with the pXRD data. The structure is essentially the same as that reported from data measured at 100K.[4]

SANS. The solutions were prepared as described above and measured in UV spectrophotometer grade, quartz cuvettes (Hellma) with a 2 mm path length. The cuvettes were placed in a temperature-controlled sample rack during the measurements. SANS measurements were performed using the D11 instrument (Institut Laue Langevin, Grenoble, France). A neutron beam, with a fixed wavelength of 6 Å and divergence of $\Delta\lambda/\lambda = 9\%$, allowed measurements over a large range in Q [$Q = 4\pi\sin(\theta/2)/\lambda$] of 0.001 to 0.3 $Å^{-1}$, by using three sample-detector distances of 1.5 m, 8m, and 39 m.

The data were reduced to 1D scattering curves of intensity vs. Q using the facility-provided software. The electronic background was subtracted, the full detector images for all data were normalized and scattering from the empty cell was subtracted. The scattering from D_2O was also measured and subtracted from the data.

SANS data was fitted to structural models in SasView software.[5] A range of models were tested for each data set and the best fitting model selected. Fit quality was determined by both the reduced chi^2 and goodness of fit by eye. Scattering length densities (SLD) were calculated for each component using the NIST neutron activation and scattering calculator.[6] An assumed density of 1.55 g/cm^3 was used. For the mixed system an average SLD based on the ratio of the two components was calculated.

H_2O:	-0.561×10^{-6} $Å^{-2}$
D_2O:	6.393×10^{-6} $Å^{-2}$
2NapFF:	2.678×10^{-6} $Å^{-2}$
2dNapdFdF:	6.861×10^{-6} $Å^{-2}$
2NapAA:	2.510×10^{-6} $Å^{-2}$
2NapFF and 2NapAA mixture:	2.560×10^{-6} $Å^{-2}$

NMR Spectroscopy. Solutions of 2NapFF and 2NapAA were prepared as described above at concentrations of 10 mg/mL in D_2O. For each sample approximately 700 μL was transferred to an NMR tube by glass pipette. NMR experiments were performed on a 400 MHz Bruker NMR spectrometer. ^{2}H experiments were performed on all solutions. ^{2}H NMR experiments were performed at a frequency of 61.4 MHz. The spectra were analysed and processed in TopSpin (version 4.0.9). The phasing of each spectrum was adjusted with a zero-order correction. ^{2}H residual quadrupolar couplings were measured in TopSpin.

Mixtures of solutions were prepared from the solutions of 2NapFF and 2NapAA in different ratios. In addition to the mixed systems, a dilution study of 2NapFF was performed in which the original gelator was diluted with D_2O to directly compare to the concentration of 2dNapdFdF in the mixed systems. The resulting mixed solutions were vortexed for 15 seconds. ^{2}H NMR experiments were performed on these solutions as described above.

Crystal growth in a magnetic field. Solutions of 2NapFF and 2NapAA were prepared as described above at a concentration of 10 mg/mL gelator each in H_2O, and starting pH adjusted to pH 10.5 ± 0.1. Once at the desired pH, solutions were then filtered using glass pipettes and cotton wool. Solutions of filtered 2NapFF and 2NapAA were then briefly mixed for 5 minutes in a 1:1 ratio. A 0.5 mL aliquot of mixed gelator solution was then added to 20 mg GdL, stirred and transferred by pipette to an NMR tube. NMR tubes were then either left undisturbed on the bench (control, without magnetic field), or immediately placed inside a 400 MHz Bruker NMR spectrometer (providing magnetic field) for 3.5 hours. After this time, optical microscope images were taken of samples with and without magnetic field using a Nikon Eclipse LV100 microscope at 5x magnification. Images were collected under polarised light. Scale bars were added to images and crystal measurements collected using the software, ImageJ. Average and standard deviation of these measurements were calculated using Microsoft Excel. A total of 10 images with and 10 images without magnetic field were created and analysed.

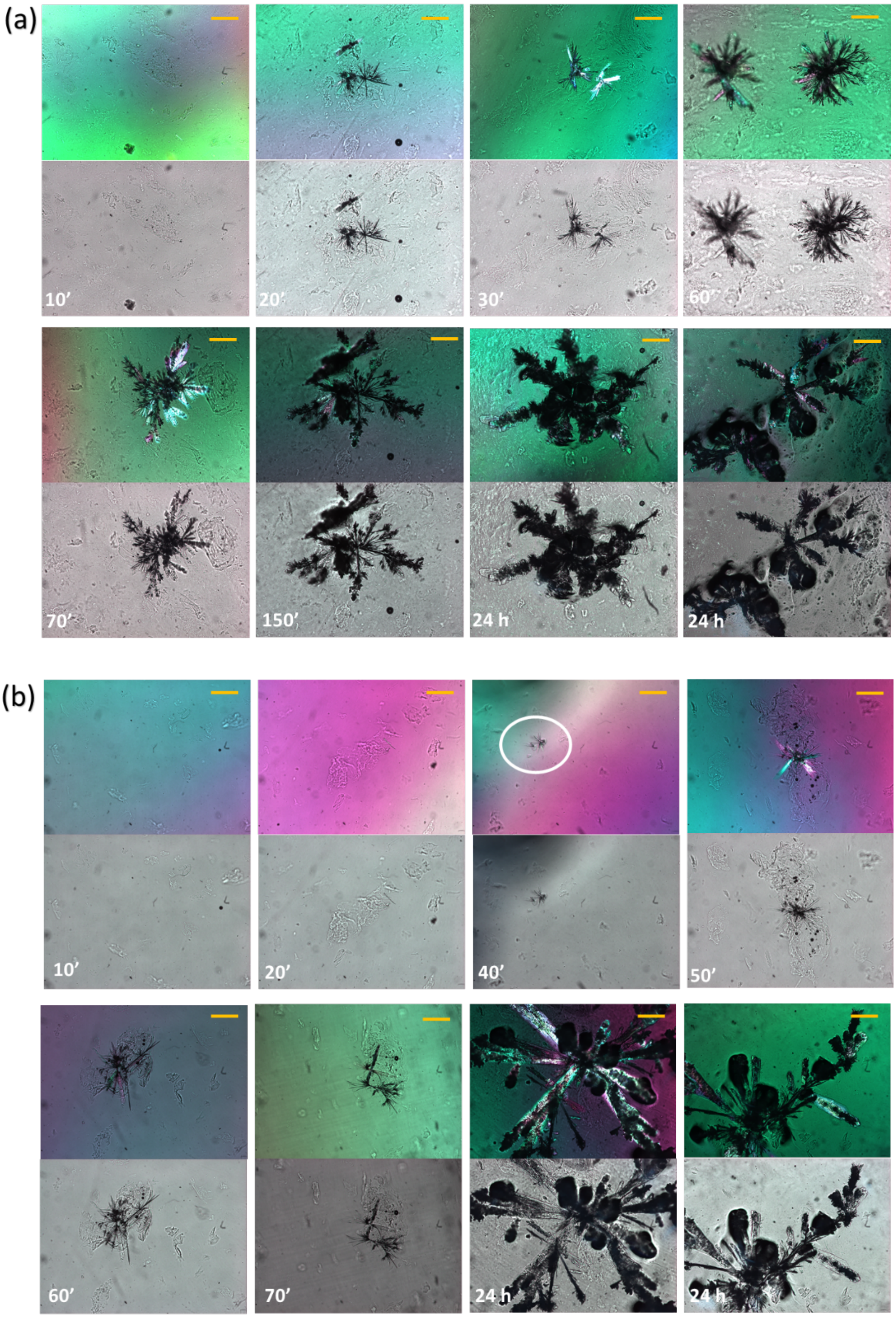

Figure S5. Optical microscope images (polarised light on the top) of sample prepared from a mixture of 2NapAA and 2NapFF in (a) H_2O and (b) D_2O over time. The scale bar represents 300 μm**.** The colors are due to the use of plastic holders used to prepare the samples; all samples are transparent.

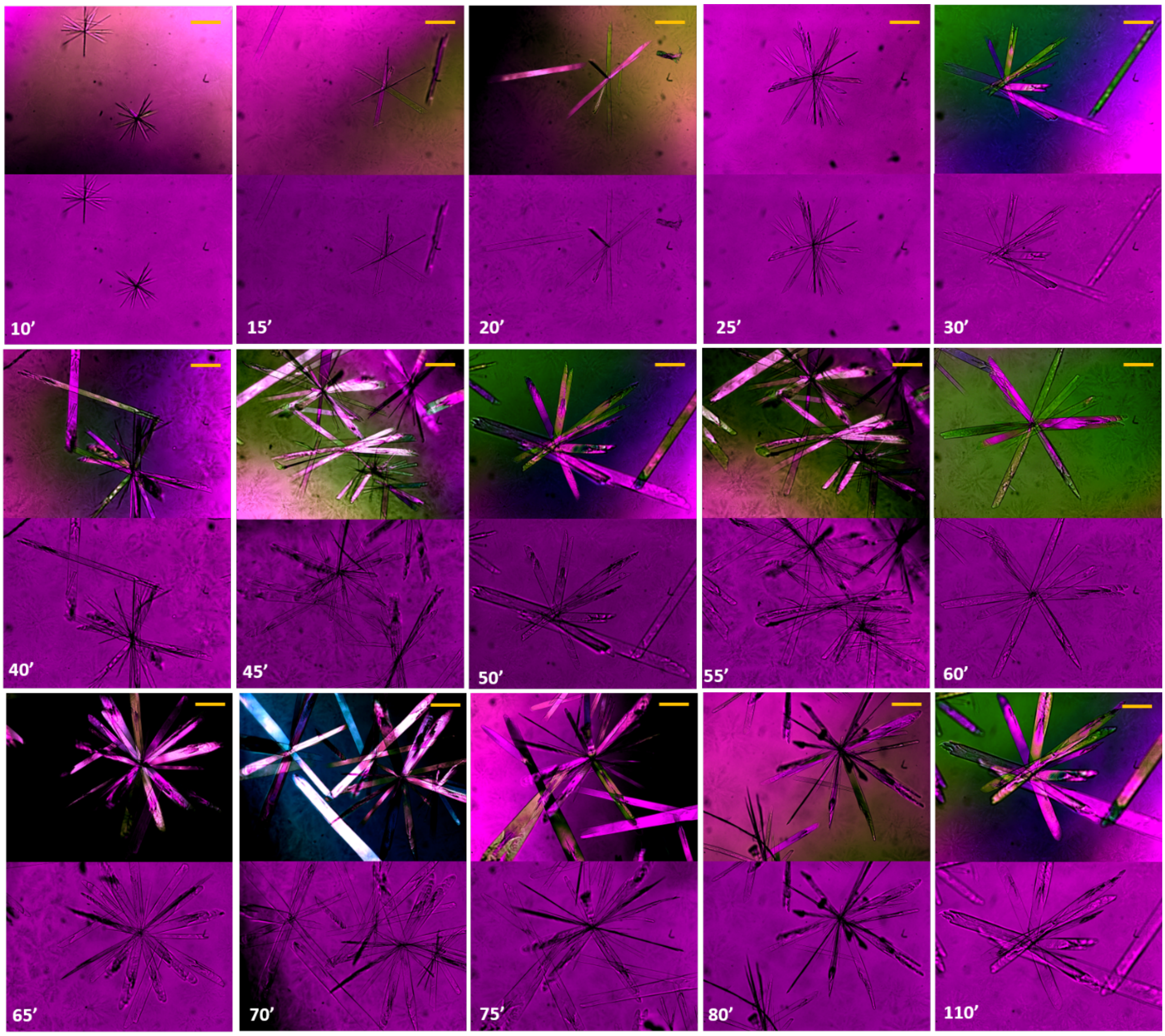

Figure S6. Optical microscope images (polarised light on the top) of crystals formed from gels of 2NapAA alone over time in H_2O. The scale bar represents 300 μm**.** The colors are due to the use of plastic holders used to prepare the samples; all samples are transparent.

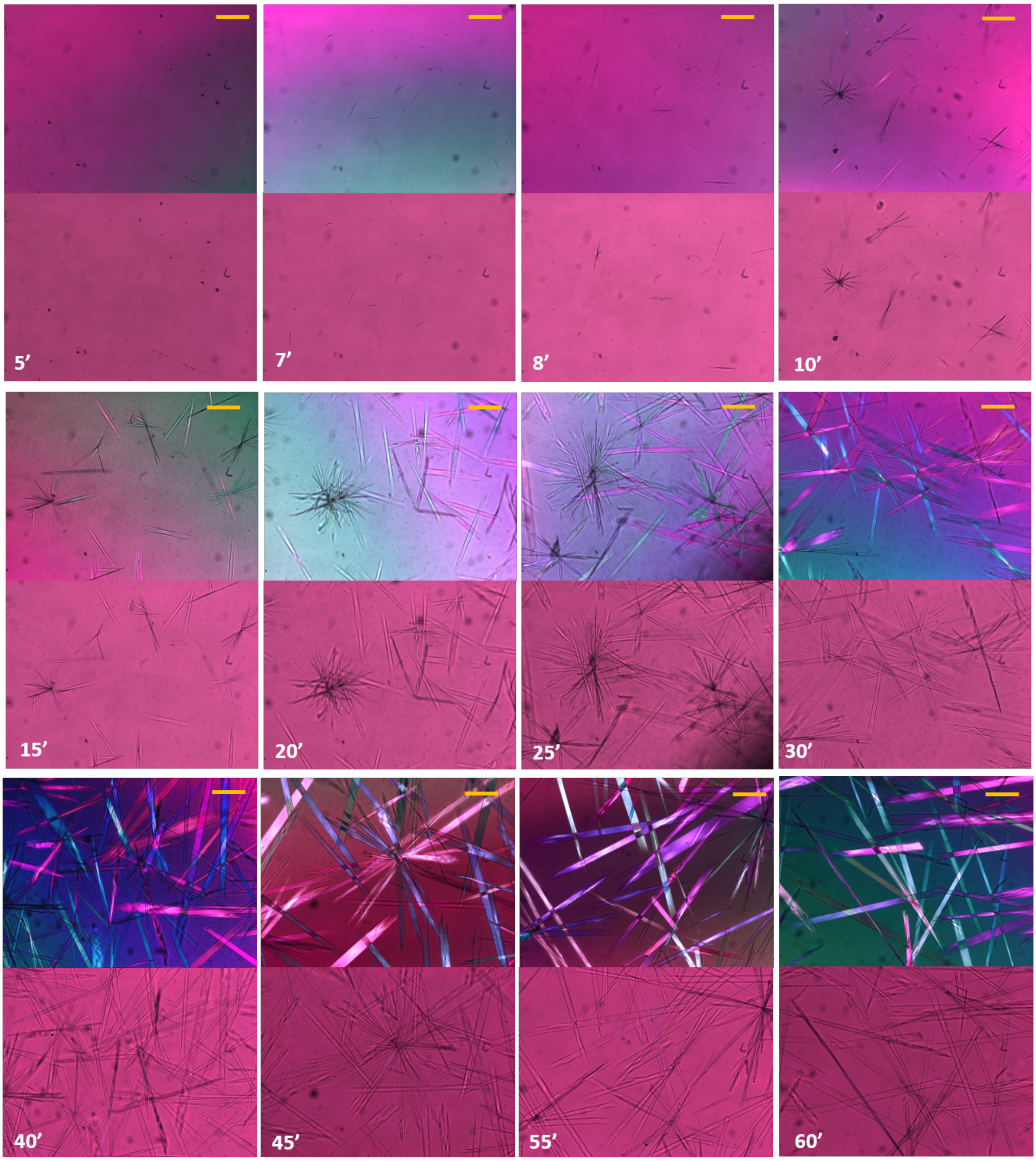

Figure S7. Optical microscope images (polarised light on the top) of crystals formed from gels of 2NapAA alone over time in D_2O. The scale bar represents 300 μm**.** The colors are due to the use of plastic holders used to prepare the samples; all samples are transparent.

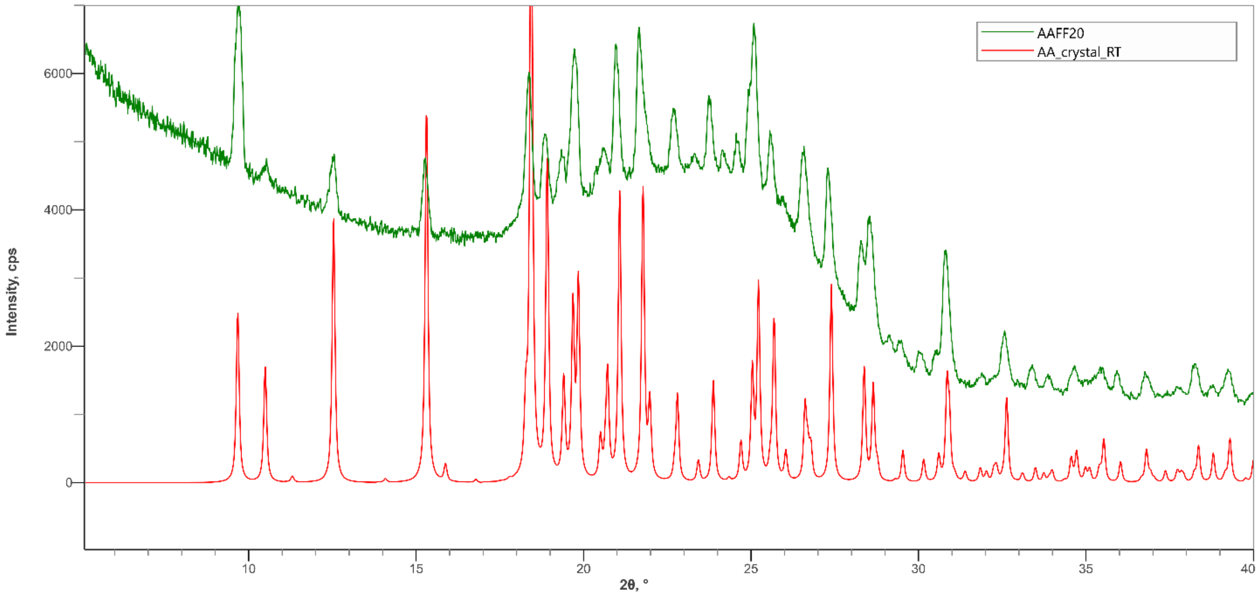

Figure S8. Powder X-ray diffraction pattern for the crystals harvested from the mixed gel (green) overlaid with the pattern calculated from the room temperature crystal structure (red). The pattern for the mixture of 2NapAA and 2NapFF has contributions from both the crystalline 2NapAA (sharp diffraction peaks) and from the non-crystalline stable gel (broad peaks). The room temperature structure, determined to facilitate comparison with pXRD data at room temperature, is essentially the same as that reported from data measured at 100K.[4]

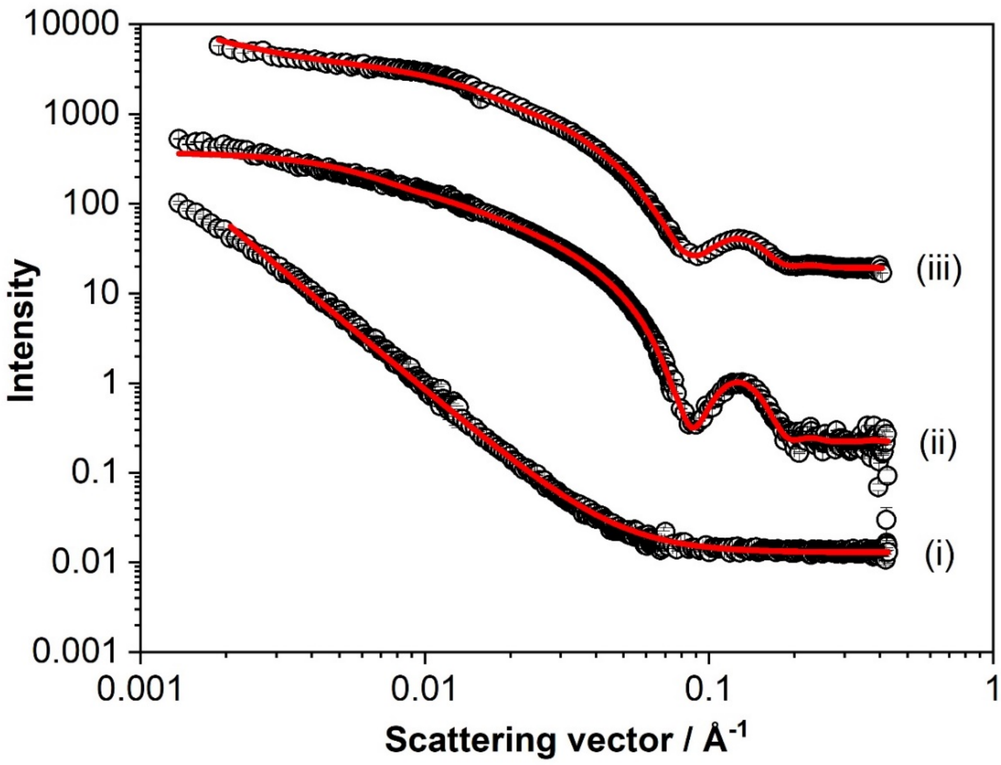

Figure S9. Small angle neutron scattering data from high pH solutions of (i) 2dNapdFdF in D_2O, (ii) 2dNapdFdF in H_2O and (iii) 2NapFF in D_2O. Black hollow circles represent experimental data and red lines represent model fits from SasView. Intensity is dimensionless because the data are offset on the same y axis. The parameters for the fitting are shown in Table S1.

Sample	2NapFF in D_2O	2dNapdFdF in H_2O	2dNapdFdF in D_2O	2NapFF:2NapAA solution	2dNapdFdF:2NapAA solution	2NapFF:2NapAA gel	2dNapdFdF:2NapAA gel
Model	Hollow cylinder + power law	Hollow cylinder	Power law	Cylinder + Power Law	Power law	Elliptical cylinder + power law	Power law
Cylinder scale	0.0049	0.0022	-	0.0005	-	0.0015	-
Cylinder scale error	0.000018	0.000016	-	0.000003	-	0.000003	-
Background	0.0467	0.0223	0.0130	0.0101	0.0161	0.0050	0.0022
Background error	0.00011	0.00034	0.00004	0.00006	0.00004	0.00004	0.00003
Radius / Å	16.46	15.72	-	41.80	-	26.70 (minor radius)	-
Radius error / Å	0.05	0.12	-	0.10	-	0.08 (minor radius)	-
Axis ratio	-	-	-	-	-	2.2	-
Axis ratio error	-	-	-	-	-	0.01	-
Thickness / Å	21.26	22.47	-	-	-	-	-
Thickness error / Å	0.08	0.19	-	-	-	-	-
Length / Å	342	813	-	343	-	3000*	-
Length error / Å	2	10	-	8	-	-	-
Power law scale	0.0002665	-	0.00000407	0.000051	0.00000225	0.000000041	0.0000002
Power law scale error	0.0000127000	-	0.0000000665	0.0000031000	0.0000000817	0.0000000015	0.0000000003
Power law	1.65	-	2.65	1.91	2.53	3.59	3.86
Power law error	0.01	-	0.00	0.01	0.01	0.00	0.00
Reduced Chi^2	9.13	2.92	2.81	1.50	1.80	4.44	10.70

Table S1. Fitting parameters obtained from SasView model fitting of the SANS data. Parameter errors are fitting errors. *Length was set to 3000 Å for the fit because the fit was going to a very large length that is far in excess of the dimensions that can be probed with SANS. This suggests that the fibres are longer than can be measured.

Choice of model for 2NapFF:2NapAA mix solution

For the 2NapFF:2NapAA mix solution at high pH, the data was best fitted to a cylinder and power law. The data for 2NapFF on its own is best fitted to a hollow cylinder as we have shown elsewhere.[3] Attempts to fit the data for the 2NapFF:2NapAA mixture to a hollow cylinder with a power law results in a cylinder with an unfeasibly small core with a thickness of 41 Å, i.e., a cylinder with no core (Figure S9 and Table S2). This fit essentially depicts the same structure obtained by the cylinder and power law fit (Figure S6 and Table S1). Fixing the radius and thickness of the hollow cylinder to those obtained for 2NapFF alone and fitting the other parameters results in an unsuitable fit, with a bump in the fit that is not present in the data (Figure S9 and Table S2). The hollow cylinder model is unsuitable, which suggests that there is a change in self-assembled structure formed by 2NapFF. The 2dNapdFdF:2NapAA mixture suggests co-assembly does not occur – if co-assembly occurred, we would expect that the scattering intensity would be significant as the 2NapAA would be forming part of the persistent structure; instead, the scattering is weak as expected from the non-scattering perdeuterated 2NapFF and the 2NapAA still forming weakly-scattering structures as when alone. These data indicate that for 2NapFF:2NapAA mix, the self-assembly of 2NapFF is influenced by the presence of 2NapAA and results in different structures, but co-assembly of the two gelators does not occur.

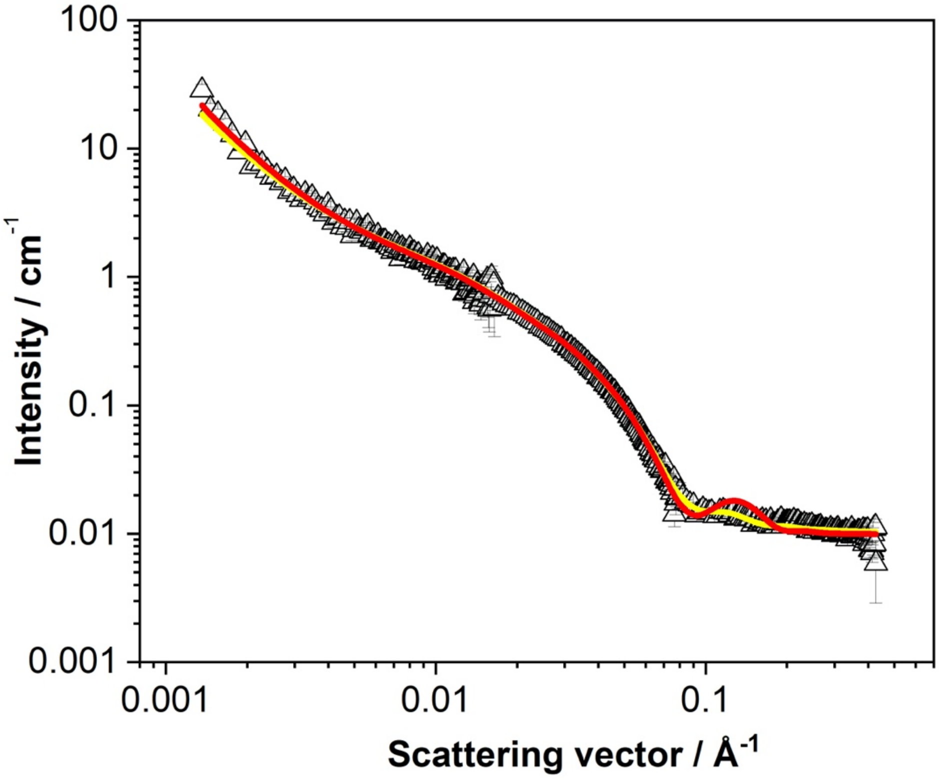

Figure S10. Small angle neutron scattering data the 2NapFF:2NapAA mix solution at high pH. Both samples were prepared in D_2O. Black triangles represent experimental data. The red and yellow lines are the model fits from SasView. The yellow line is the model fit for a hollow cylinder combined with a power law. The red line is the model fit for a hollow cylinder (fixed radius and thickness) combined with a power law. The parameters for the fitting are shown in Table S1.

Model	Cylinder + Power Law	Hollow cylinder + power law (fitted)	Hollow cylinder + power law (fixed radius and thickness)
Cylinder scale	0.0005	0.0005	0.0007
Cylinder scale error	0.000003	0.000003	0.000003
Background	0.0101	0.0101*	0.0101*
Background error	0.00006	-	-
Radius / Å	41.80	0.01	16.00*
Radius error / Å	0.10	481.51	-
Thickness / Å	-	41.95	21.00*
Thickness error / Å	-	481.55	-
Length / Å	343	345	349
Length error / Å	8	8	7
Power law scale	0.000051	0.000064	0.000022
Power law scale error	0.000003	0.000002	0.000001
Power law	1.91	1.87	2.06
Power law error	0.01	0.01	0.01
Reduced Chi^2	1.50	1.55	4.73

Table S2. Fitting parameters for different fits to the 2NapFF:2NapAA mix at high pH. The data demonstrate why the cylinder + power law model is most appropriate for this data. *Represents values that were fixed and not fitted.

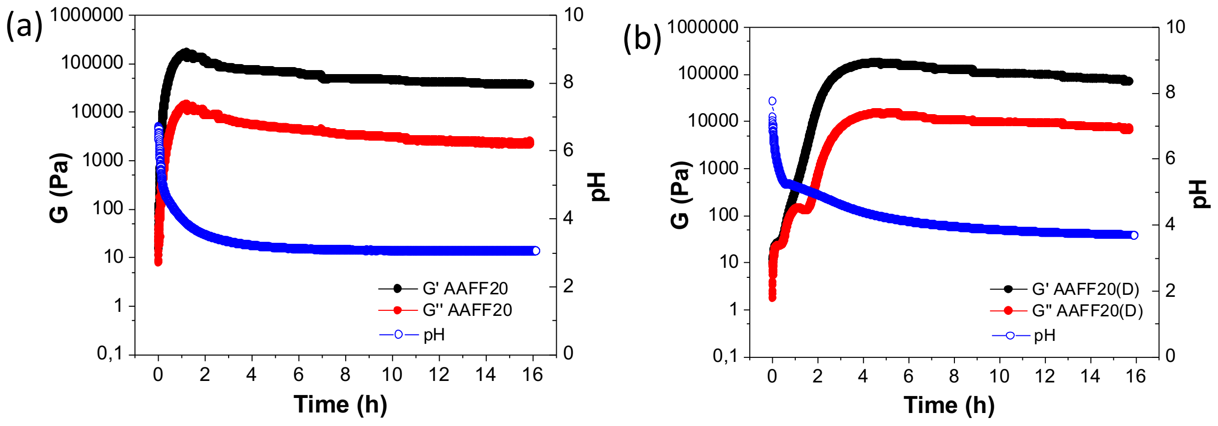

Figure S11. Comparison of the rheology and pH change in (a) H_2O and (b) D_2O for the mixture of 2NapAA and 2NapFF. The rheological data are as for Figure 3 but shown over a longer time. In both (a) and (b), the black data shows G′, the red data shows G″ (left axis) and the blue data shows pH (right axis).

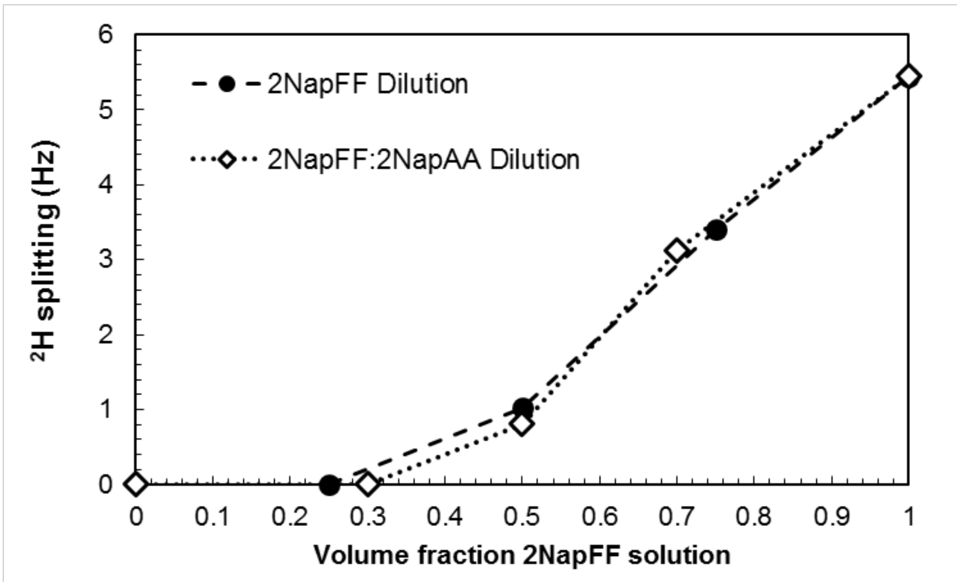

Figure S12. Plot of residual quadrupolar coupling for ^{2}H in solutions of 2NapFF diluted with either a solution of 2NapAA or D_2O in different ratios.

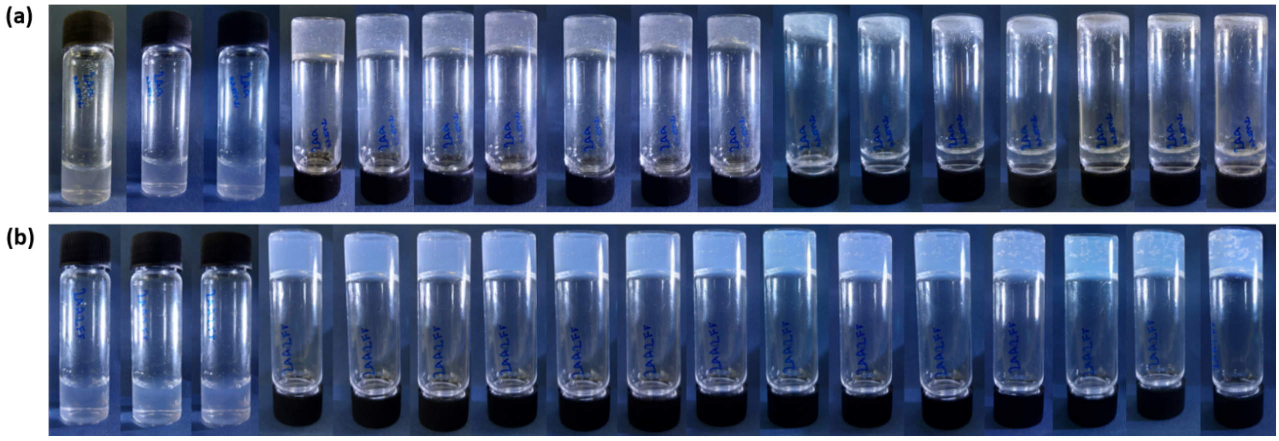

Figure S13. (a) 2NapAA (5 mg/mL) single and (b) 2NapAA (5 mg/mL) and 2NapFF (2.5 mg/mL) mixed systems after addition of 40 mg GdL at times 0, 5, 10, 15, 20, 25, 30, 35, 40, 45, 50, 55, 60, 90, 120, 150 and 180 minutes. Crystals began to form within the first 5 minutes. Both samples became invertible at 15 minutes. The 2NapAA single system began to flow after 30 minutes (not clearly visible in photos until 45 minutes) with almost all liquid reaching the bottom of the vial after 60 minutes. The mixed system remained a gel for the full 3 hours with crystals continuing to grow throughout. The sample volume was 2 mL and the vials had diameters of 16 mm.

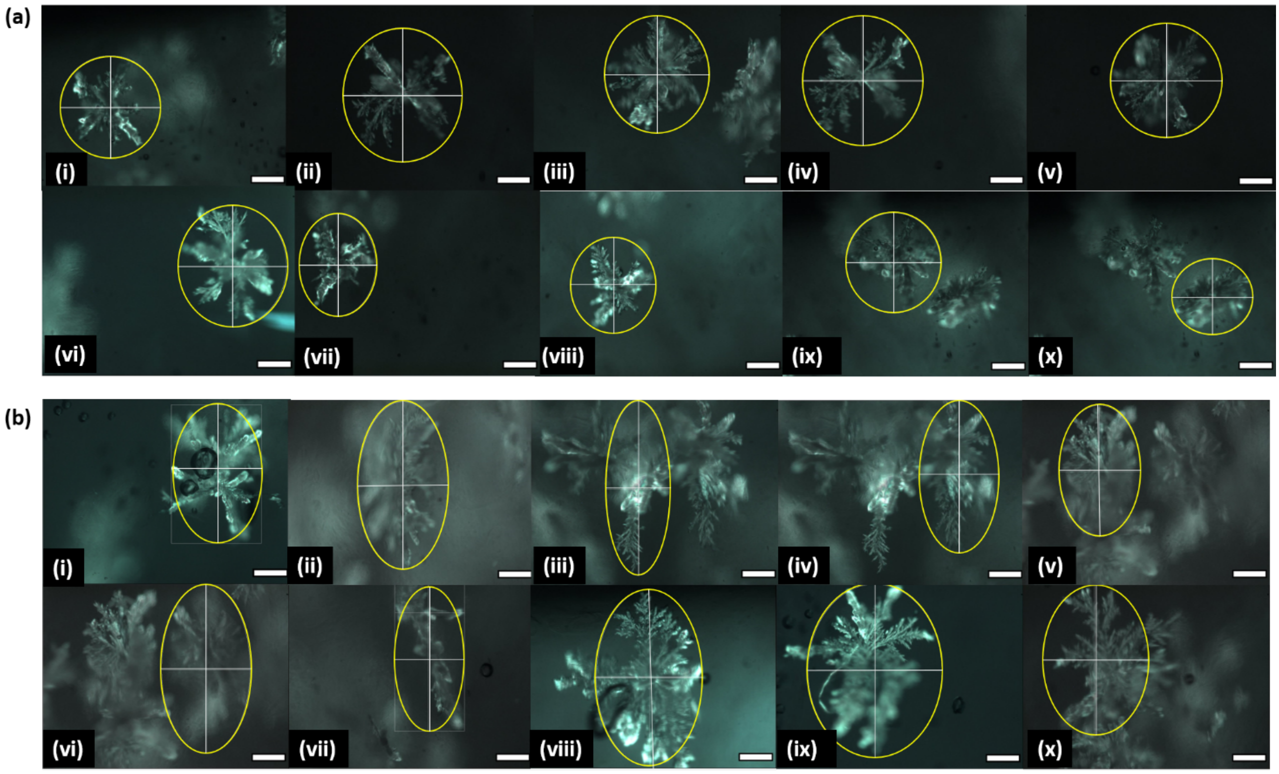

Figure S14. Crystal growth of multicomponent system in magnetic field. The lengths of the 2NapAA crystallites mixed with 2NapFF were measured from optical microscope images collected under polarised light using ImageJ. The mean lengths along the x- and y-axes (white horizontal and vertical lines respectively) were evaluated from crystals grown (a) without and (b) with the presence of a magnetic field. 10 measurements were collected and analysed for each. The magnetic field was positioned along the x-axis in all cases, meaning elongation occurs in the y-direction. The yellow shapes highlight the spherical and elongated structures of the crystals without and with magnetic alignment respectively. Scale bars represent 300 μm.

Presence of Magnetic Field	n	Mean Y (mm)	Mean X (mm)	Mean Y/X (mm)	Standard Deviation (mm)
No Magnetic Field	10	0.609	0.559	1.092	0.104
Magnetic Field	10	0.923	0.524	1.849	0.418

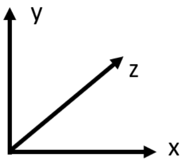

Table S3. The lengths of the 2NapAA crystals mixed with 2NapFF were measured from optical microscope images collected under polarised light using ImageJ. The mean lengths along the x- and y-axes was evaluated from crystals grown with and without the presence of a magnetic field. 10 images were collected and analysed from 3 separate samples for each condition. Errors were calculated from the mean values. The magnetic field was positioned along the x-axis in all cases, meaning elongation occurs in the y-direction.

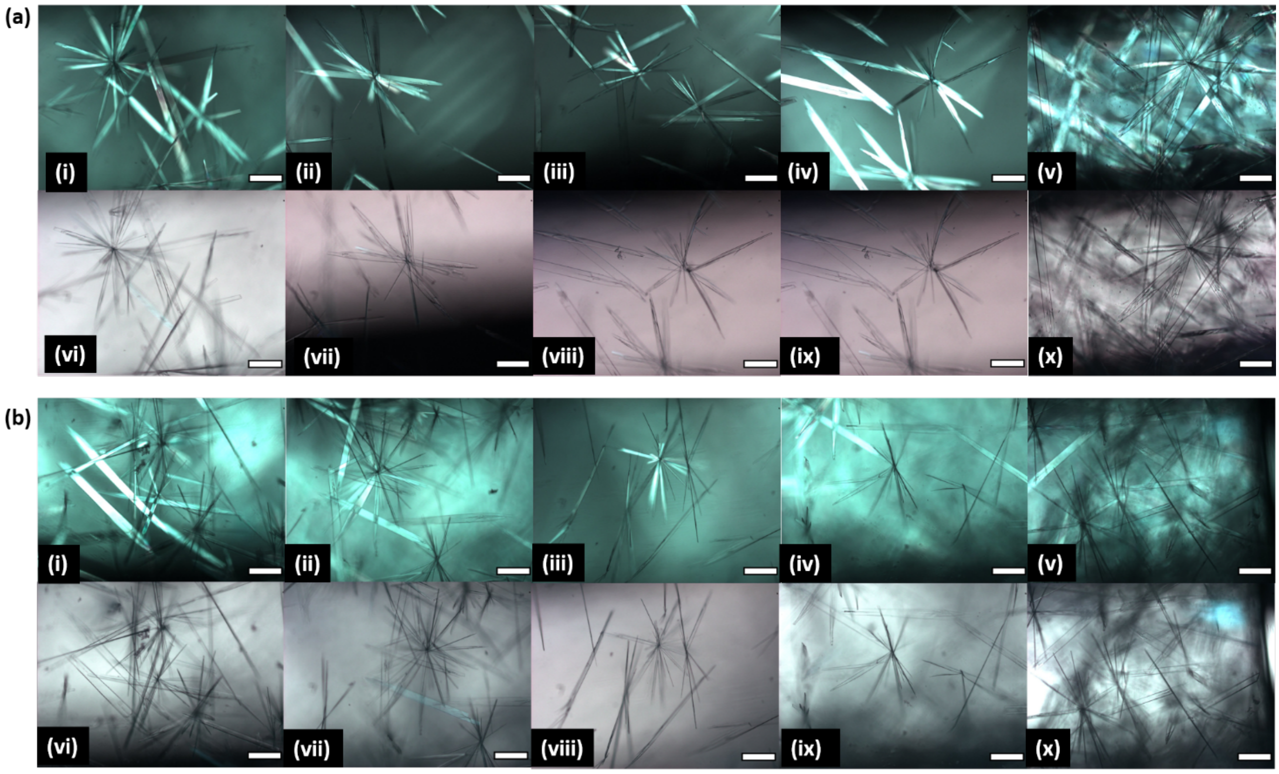

Figure S15. Optical microscope images of crystals formed from 2NapAA alone in H_2O (a) without and (b) with the presence of a magnetic field. (i)-(v) were collected under polarised light and (vi)-(x) were collected under non-polarised light. No alignment or considerable change in crystal morphology was observed in samples prepared within the magnetic field compared to the control samples without a magnetic field. The scale bars represent 300 μm**.**

References

1. Chen, L.; Revel, S.; Morris, K.; C. Serpell, L.; Adams, D. J., Effect of Molecular Structure on the Properties of Naphthalene−Dipeptide Hydrogelators. Langmuir **2010,** 26 (16), 13466-13471.
2. http://cssp.chemspider.com/Article.aspx?id=924.
3. Draper, E. R.; Dietrich, B.; McAulay, K.; Brasnett, C.; Abdizadeh, H.; Patmanidis, I.; Marrink, S. J.; Su, H.; Cui, H.; Schweins, R.; Seddon, A.; Adams, D. J., Using Small-Angle Scattering and Contrast Matching to Understand Molecular Packing in Low Molecular Weight Gels. Matter **2020,** 2 (3), 764-778.
4. Houton, K. A.; Morris, K. L.; Chen, L.; Schmidtmann, M.; Jones, J. T. A.; Serpell, L. C.; Lloyd, G. O.; Adams, D. J., On Crystal versus Fiber Formation in Dipeptide Hydrogelator Systems. Langmuir **2012,** 28 (25), 9797-9806.
5. www.sasview.org/.
6. https://www.ncnr.nist.gov/resources/activation/ Accessed 19/04/21.
